# Technical complications with tooth-supported fixed dental prostheses (FDPs) of different span lengths: an up to 15-year retrospective study

**DOI:** 10.1186/s12903-023-03121-9

**Published:** 2023-06-15

**Authors:** Ali Alenezi, Sarah Aloqayli

**Affiliations:** 1 Department of Prosthodontics, College of Dentistry, Qassim University, P.O. Box 6700, Buraydah, Saudi Arabia; 2 Intern, College of Dentistry, Qassim University, Buraydah, Saudi Arabia

**Keywords:** Survival rate, Failures, Technical complications, Fixed dental prosthesis, Retrospective clinical study

## Abstract

**Backgrounds:**

Long-span dental bridges may cause excessive load on abutment teeth and the periodontal area, which may lead to bridge fractures or periodontal problems. However, some reports have revealed that short- and long-span bridges can provide a similar prognosis. This clinical study aimed to investigate the technical complications associated with fixed dental prostheses (FDPs) of different span lengths.

**Methods:**

All patients with previously cemented FDPs were clinically examined during their follow-up visits. Several data related to FDPs were registered, such as design, material type, location, and type of complication. The main clinical factors analyzed were technical complications. Life table survival analyses were performed to calculate the cumulative survival rate of FDPs when technical complications were detected.

**Results:**

The study examined 229 patients with a total number of 258 prostheses and an average of 98 months of follow-up. Seventy-four prostheses suffered from technical complications, and the most common complication was ceramic fracture or chipping (*n* = 66), while loss of retention occurred in 11 prostheses. The long-term evaluation of long-span prostheses revealed a significantly higher technical complication rate compared to short-span prostheses (*P* = ,003). The cumulative survival rate for short-span FDPs was 91% in year 5, 68% in year 10, and 34% in year 15. For long-span FDPs, the cumulative survival rate was 85% in year 5, 50% in year 10, and 18% in year 15.

**Conclusion:**

Long-span prostheses (5 units or more) can be associated with a higher technical complication rate compared to short-span prostheses after long-term evaluation.

**Supplementary Information:**

The online version contains supplementary material available at 10.1186/s12903-023-03121-9.

## Introduction

Teeth loss is caused by different reasons. Some studies have reported that tooth loss may affect patients psychologically and have a negative impact on their quality of life [1, 2]. Fortunately, different treatment options are available for replacing missing teeth, such as removable fixed prostheses and implant restorations. For each treatment option, evidence-based clinical data are required to evaluate the survival and complication rates for each treatment during the decision-making process.

In recent decades, replacing missing teeth with implant restorations has emerged as the preferred treatment option in most cases of missing teeth. Many reports have revealed a high success rate and patient satisfaction with implant-supported reconstructions [3]. However, implant treatment may not be an option for different reasons, such as in the case of severe bone resorption or when the surgery is contraindicated for the patient for medical reasons [4, 5]. In addition, many patients still choose to be treated with fixed dental prostheses (FDPs) to avoid the surgical procedures required for implant treatment [6]. Several studies have revealed that FDP treatment can offer patients exceptional satisfaction in replacing their missing teeth [7, 8]. Although treatment with FDPs may include the removal of sound tooth structure during tooth preparation, many reports have shown that it can provide a high success rate in the long term [9, 10]. It is believed that good treatment outcomes are influenced by different factors, such as proper treatment plans and patients maintaining good oral hygiene.

Fixed dental prostheses are made from several types of materials. Metal-based FDPs have been considered the gold standard for many decades due to their reliability and clinical use [11]. Numerous reports have revealed a high success rate for conventional porcelain-fused-to-metal (PFM) restorations after a long-term evaluation [12, 13]. However, some researchers have investigated more biocompatible materials, such as lithium disilicate and zirconium oxide ceramics, in order to overcome some the limitations of metal-based FDPs [14, 15]. one of the main limitations of metal-based FDPs is the presence of gray metal framework that make the process of fabricating natural esthetics difficult [16]. For this purpose, a new framework based on ceramic materials was introduced to improve esthetics and enhance restoration biocompatibility [17, 18]. However, due to their low mechanical properties, non-oxide-based ceramics, rather than FDPs, were recommended mainly for the anterior region or as a single crown restoration material [17, 19, 20].

Several reports have discussed the failure rate based on different types of complications [21–23]. Technical complications, such as fracture or deboning, are believed to be influenced by the treatment plan and clinician work [24]. For instance, several studies have suggested that bridge design contributes greatly to the risk of these complications. Failure to provide the prosthesis with the proper design criteria for esthetics and function may result in failed treatment.

Technical complications have been investigated extensively in the literature and were reported to be the most common type of complications with FDPs [25, 26]. In a review published by Jokstad [27], the estimated risk of FDP loss over 10 years can be caused by abutment fracture (2.1%), loss of retention (6.4%), or material fractures (3.2%). A systematic review and meta-analysis were published by Pjetursson et al. [28], in which around 2,906 fixed prostheses made of metal-ceramic and all-ceramic materials with a mean follow-up of at least 3 years were investigated. They found significantly more framework fractures for FDPs made of all-ceramics compared to metal-ceramic FDPs [28]. Meanwhile, chipping of the veneering ceramic was reported as the most common technical complication, with all-ceramic FDPs occurring in 12.7% after a 3-year observation period [28]. For metal-ceramic FDPs, chippings, or fractures of the ceramic are frequently reported, particularly in the first year in function, but some reports have found that the rate of this complication may slightly decrease after that [29].

Understanding the causes of clinical complications associated with fixed prosthodontics may improve the clinician’s experience to select the best treatment plan for his patients. In addition, patients will have realistic expectations of treatment outcomes and post-treatment care. For the FDP treatment plan, Ante’s law states that the surface area of abutment teeth roots should be equal to or greater than the area that will be replaced with pontics [30]. This recommendation enables the abutment teeth to withstand occlusal forces during function. Excessive loads on the prosthesis may lead to technical complications or negative tissue responses. Meanwhile, some reports have claimed that the longer the span (5-unit or more), the greater the risk of FDPs flexing [10, 21, 31]. Therefore, many clinicians try to ovoid restoring the edentulous area, which consists of more than two teeth with FDPs. Several studies have investigated the influence of FDP span length on complication rates. A recently published clinical study reported that the complication rate of FDP will increase with each increase in pontics numbers [32]. Similar findings were reported in another clinical study, in which higher complication rates for long-span FDPs made of cobalt-chromium (Co-Cr) were compared to short-span FDPs [33]. Meanwhile, a 20-year retrospective study published by De Backer et al. [34] revealed better results for short-span versus long-span FDPs. Furthermore, numerous studies have reported that chipping is a major problem in FDPs of 5 units or more [35–37]. Meanwhile, a clinical study by Ferrario et al. revealed a similar risk of failure for long-span FDPs when they were placed in the molar or anterior region [38, 39]. Therefore, some researchers believe that FDP should be indicated only in the short-span edentulous area with the presence of proper periodontal support that will help the prosthesis against occlusal forces.

It is believed that a long-span bridge may cause an excessive load on the bridge and the periodontal area, which may lead to fractures or periodontal problems. However, some reports have revealed that short- and long-span bridges can provide similar prognoses. This clinical study aimed to investigate the technical complications associated with fixed dental prostheses of different span lengths. Based on span length, the FDPs were classified into two main groups: short-span FDPs (3 or 4 units) and long-span FDPs (5 units or more). The null hypotheses were that no difference would be found in complication rates between short- and long-span FDPs.

## Materials and methods

The study plan was reviewed and approved by the ethics committee in the faculty of dentistry at Qassim University (Registration number ST/6094/2021) to investigate the technical complications related to cemented FDPs. In this study, all patients with previously cemented FDPs were examined clinically during their latest visits to the prosthodontic department in the collage of dentistry at Qassim university. Some of the patients visited the clinics complaining from their existing FPDs while other came for other treatment plans. The study included patients aged 18 years and older who were able to sign informed consent. Patients with removable prostheses, Implant-supported FDPs, and Cantilever FDPs were not included. Meanwhile, patients with parafunctional habits were no excluded from this study. This study included 229 patients with a mean age of 58.2 years, of whom 123 were male and 106 were female. During the follow-up visits, full clinical and radiographical exams were performed on the patients to evaluate the existing prosthesis and report any complications associated with it. All clinical examinations were conducted by the authors of this study and took place at dental clinics at the College of Dentistry at Qassim University from July 2022 until January 2023. Before the examination, all the patients were requested to complete and sign informed consent forms. The patients’ anonymity was preserved during the study. Following the clinical examinations, several data related to the FDP were registered, such as the design of the bridge, type of material, location, and type of complication.

The main clinical factor analyzed was technical complications. Technical complications included framework fracture, minor or major fracture of the veneering porcelain, tooth fracture, mobility, abutment loss, loss of retention, and marginal discoloration. The prostheses were further divided into groups based on gender, span length, material type, location (maxilla or mandible), and area (anterior, anterior–posterior, posterior, or cross-arch). In addition, the examined FDPs were classified into two groups based on the materials used: PFM and all ceramic materials. Although different ceramic materials of different compositions can be used to fabricate, it was decided in this study to consider all different types of all-ceramic FDPs as one group. Following the clinical examinations, additional appointments were arranged for patients who needed additional treatment related to their FDPs.

The main inclusion criteria included patients older than 18 years and able to sign the informed consent form. Only conventional FDPs that were tooth-supported on both sides were included. Removable prostheses or implant-supported FDPs were not included in this study. Meanwhile, a follow-up time of 3 years or more was requested for all the prostheses to be considered in the study. The reporting guidelines of the Strengthening the Reporting of Observational Studies in Epidemiology (STROBE) checklist [40] were done in this study.

The data were statistically examined using the Statistical Package for the Social Sciences (SPSS) version 28 software (SPSS Inc., Chicago, IL, USA). Kaplan–Meier survival analysis was used to investigate FDPs’ survival rate based on the cumulative incidence of technical complications. Life table survival analyses were done to calculate the cumulative survival rate of FDPs when technical complications were detected. The Mann–Whitney U test was used to test the significant differences between the different evaluated groups. The significance level was set at *p* ≤ 0.05.

## Results

Table 1 summarizes all the findings obtained from the examined patients. Of these patients, a total of 258 prostheses were evaluated, with an average of 98 months of follow-up. During the clinical examination, 75 prostheses had technical complications (28.7%). The most common complication was porcelain fracture or chipping (*n* = 66), while loss of retention occurred in 11 prostheses. Three of the prostheses showed both of these complications.

**Table 1:** Descriptive data of the FDPs included in the study, with follow-up time between the different factors. The statistical unit is the prosthesis, not the patient

Group		Number of FDPs (%)	Number of FDPs (%) with technical complications	Mean observation period (months) ± SD	*P*- value
**Gender**	**Male**	**136 (52.7%)**	**34 (25%)**	**96.5 ± 83.4**	**0.129**
	**Female**	**122 (47.3%)**	**41(33.6%)**	**95.8 ± 82.2**	
**Span length**	**Short**	**197 (76.4%)**	**48 (24.3%)**	**93.92 ± 83.03**	**0.003**
	**Long**	**61(23.6%)**	**27 (44.2%)**	**105.37 ± 84.29**	
**Jaw**	**Maxilla**	**141(54.7%)**	**42 (29.8%)**	**93 ± 81.49**	**0.781**
	**Mandible**	**117(45.3%)**	**33 (28.2%)**	**100.1 ± 85.60**	
**FPD location**	**Anterior**	**11 (4.3%)**	**5 (45.45%)**	**93.8 ± 79.74**	**0.263**
	**Posterior**	**139 (53.9%)**	**34 (24.6%)**	**92.80 ± 82.90**	
	**Anterior/posterior**	**60 (23.3%)**	**19 (31.6%)**	**87.4 ± 78.61**	
	**Cross-arch**	**48 (18.6%)**	**17 (35.4%)**	**119.9 ± 89.17**	
**Type of material**	**Full ceramic**	**24 (9.3%)**	**5 (20.8%)**	**74.5 ± 61.2**	**0.352**
	**PFM**	**234 (90.7%)**	**70 (29.9%)**	**98.90 ± 85.02**	
**Total**		**258**	**75**		

**Fig. 1:**
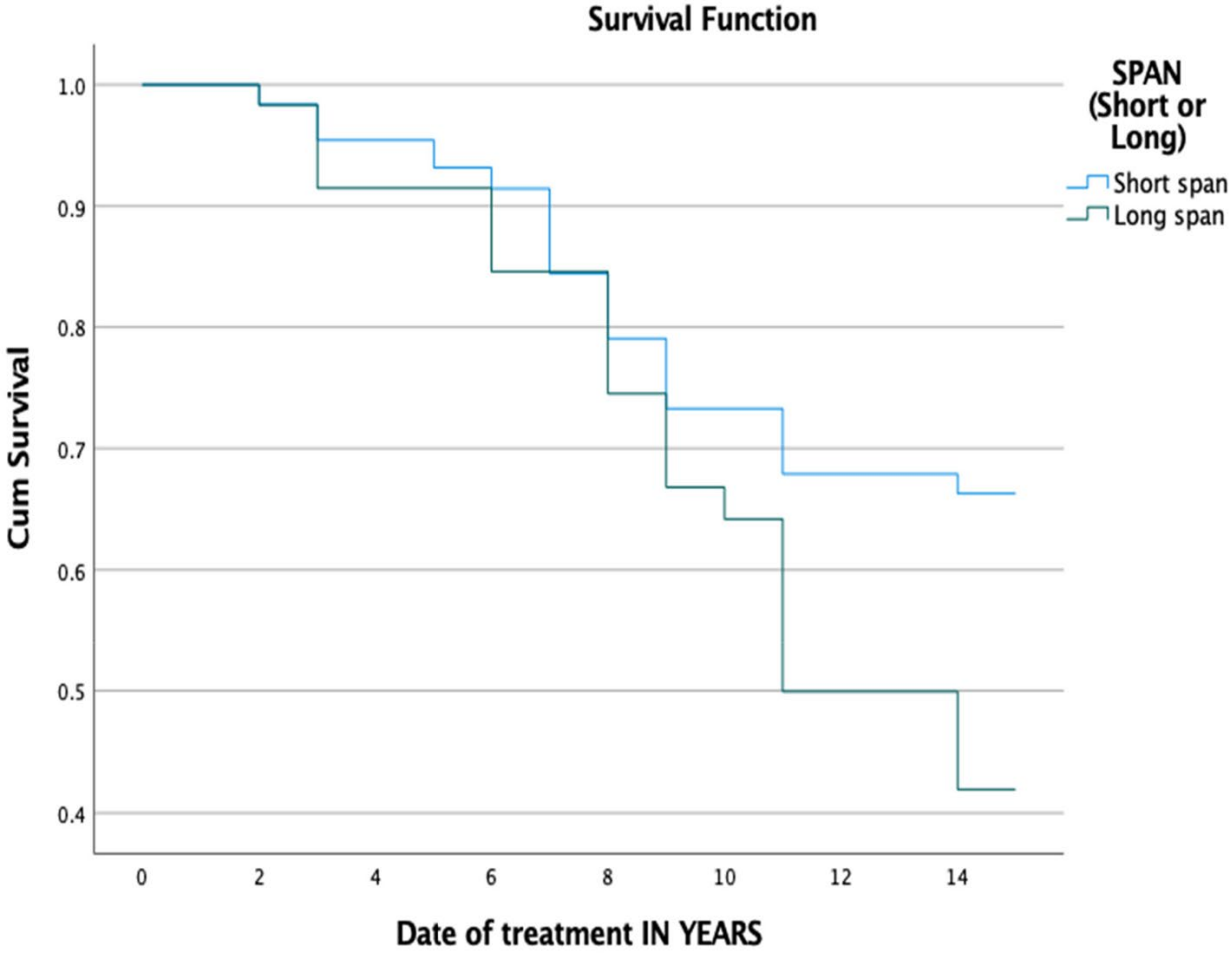
Kaplan–Meier survival function of FDPs for primary outcome ‘technical complications’ based on span length, (*P*-value = .003)

**Fig. 2:**
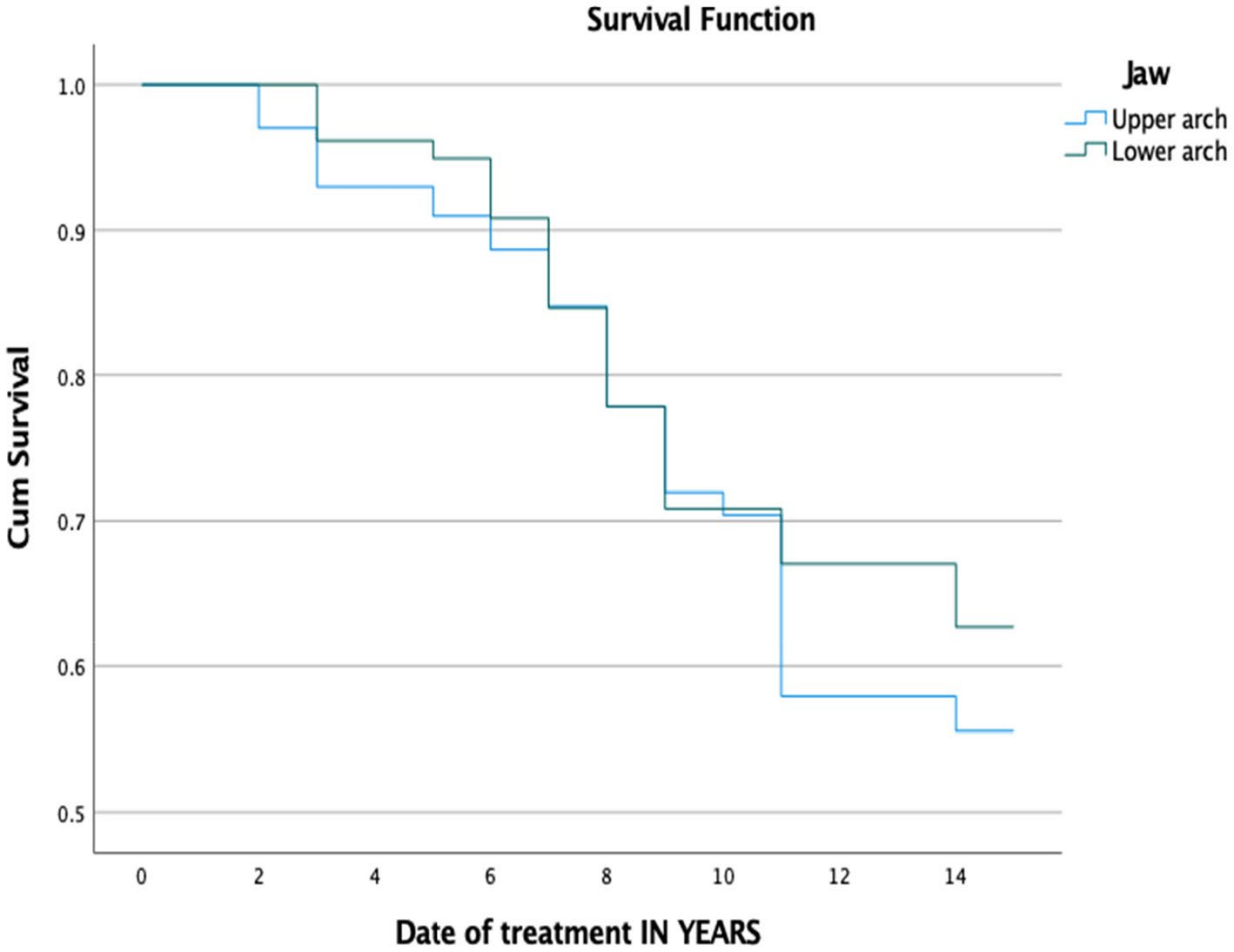
Kaplan–Meier survival function of FDPs for primary outcome ‘technical complications’ based in upper and lower jaws, (*P*-value = 0.31)

Female patients were associated with a higher rate (33.6%) of technical complications compared to male patients (25%), but the differences were not statistically significant (*P* = ,129). All 258 prostheses were categorized based on span length, location, and type of material. Most of the examined prostheses (76% of the total) were short-span (3 or 4 units), while the remaining prostheses (23.6%) were long-span FDPs that consisted of 5 units or more. The long-term evaluation of the long-span FDPs revealed a significantly higher technical complications rate compared to short-span FDPs (*P* = ,003) after a 15-year follow-up (Fig. 1). Meanwhile, no significant differences were found (*P*-value = 0.31) between upper and lower jaws regarding the occurrence of technical complications (Fig. 2). Tables 2 and 3 show the life-table survival analysis of FDPs placed in upper and lower jaws. The area of the dental prosthesis (anterior, anterior–posterior, posterior, or cross-arch) was found to have no significant impact on the rate of technical complications (*P* = ,263). The majority of evaluated FDPs were made of PFM (90.7%) and showed a higher complication rate than full ceramic, but that was not statistically significant (*P* = ,352).

**Table 2:** Life-table survival analysis showing the cumulative survival rate of short span FDPs when it comes to occurrence of technical complications

Interval Start Time (Years)	Number Entering Interval	Number Withdrawing during Interval	Number Exposed to Risk	**FDPs** Failure	Survival Rate within each Intrerval – ISR (%)	Cumulative Proportion Surviving at End of Interval	Std. Error (%)
1	197	19	187.500	3	0.98	0.98	0.01
2	175	16	167.000	5	0.97	0.95	0.02
3	154	23	142.500	0	1.00	0.95	0.02
4	131	10	126.000	3	0.98	0.93	0.02
5	118	20	108.000	2	0.98	0.91	0.02
6	96	9	91.500	7	0.92	0.84	0.03
7	80	4	78.000	5	0.94	0.79	0.04
8	71	5	68.500	5	0.93	0.73	0.04
9	61	1	60.500	0	1.00	0.73	0.04
10	60	11	54.500	4	0.93	0.68	0.05
11	45	1	44.500	0	1.00	0.68	0.05
12	44	1	43.500	0	1.00	0.68	0.05
13	43	1	42.500	1	0.98	0.66	0.05
14	41	1	40.500	0	1.00	0.66	0.05
15	40	27	26.500	13	0.51	0.34	0.07

**Table 3:** Life-table survival analysis showing the cumulative survival rate of long span FDPs when it comes to occurrence of technical complications

Interval Start Time (Years)	Number Entering Interval	Number Withdrawing during Interval	Number Exposed to Risk	**FDPs** Failure	Survival Rate within each Intrerval – ISR (%)	Cumulative Proportion Surviving at End of Interval	Std. Error (%)
1	61	2	60.000	1	0.98	0.98	0.02
2	58	1	57.500	4	0.93	0.91	0.04
3	53	5	50.500	0	1.00	0.91	0.04
4	48	6	45.000	0	1.00	0.91	0.04
5	42	4	40.000	3	0.93	0.85	0.05
6	35	1	34.500	0	1.00	0.85	0.05
7	34	1	33.500	4	0.88	0.75	0.07
8	29	0	29.000	3	0.90	0.67	0.07
9	26	1	25.500	1	0.96	0.64	0.07
10	24	3	22.500	5	0.78	0.50	0.08
11	16	2	15.000	0	1.00	0.50	0.08
12	14	1	13.500	0	1.00	0.50	0.08
13	13	1	12.500	2	0.84	0.42	0.09
14	10	0	10.000	0	1.00	0.42	0.09
15	10	6	7.000	4	0.43	0.18	0.09

**Fig. 3:**
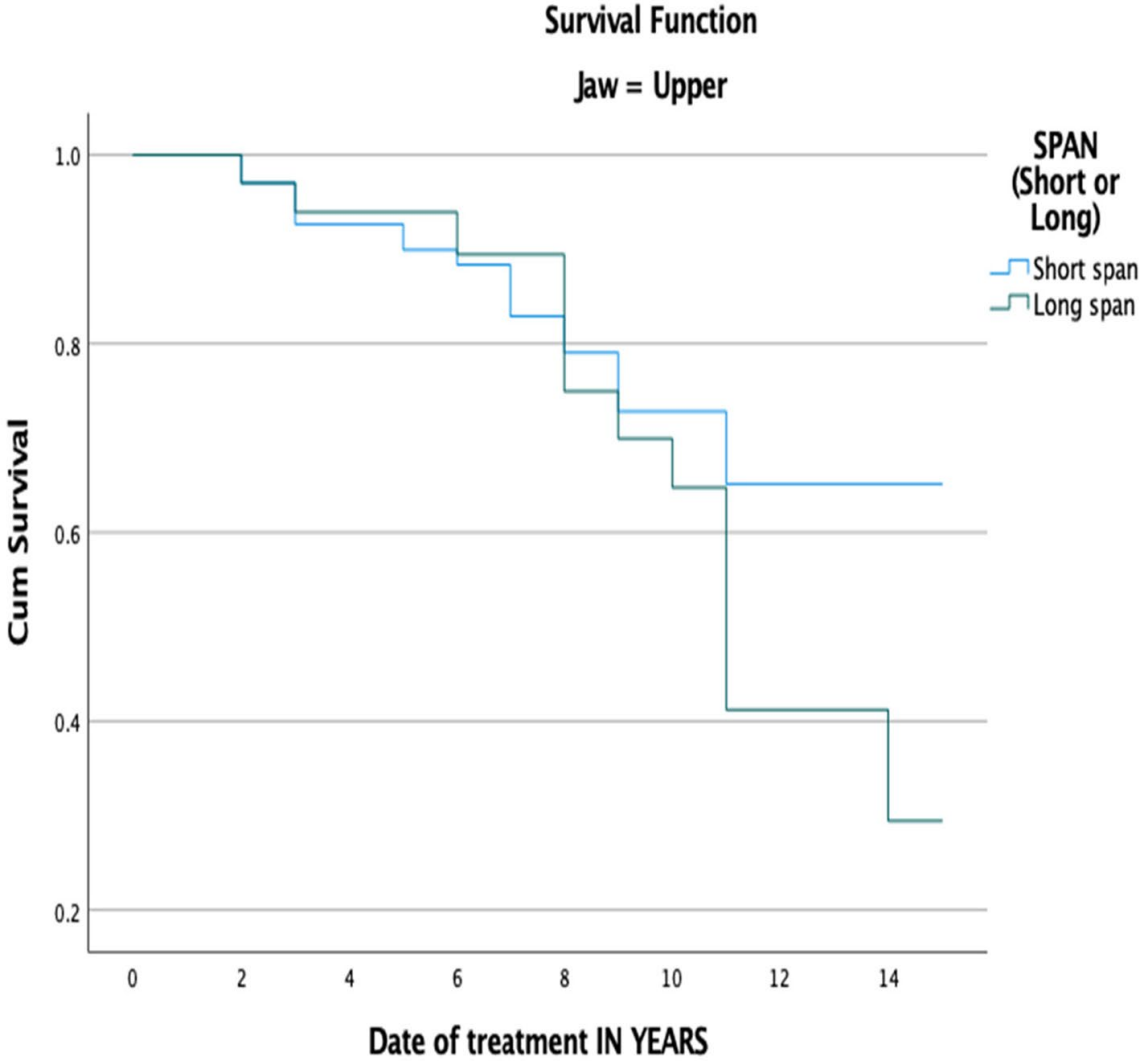
Kaplan–Meier survival function of FDPs in the upper jaw for primary outcome ‘technical complications’ based on span length, (*P*-value = .048)

**Fig. 4:**
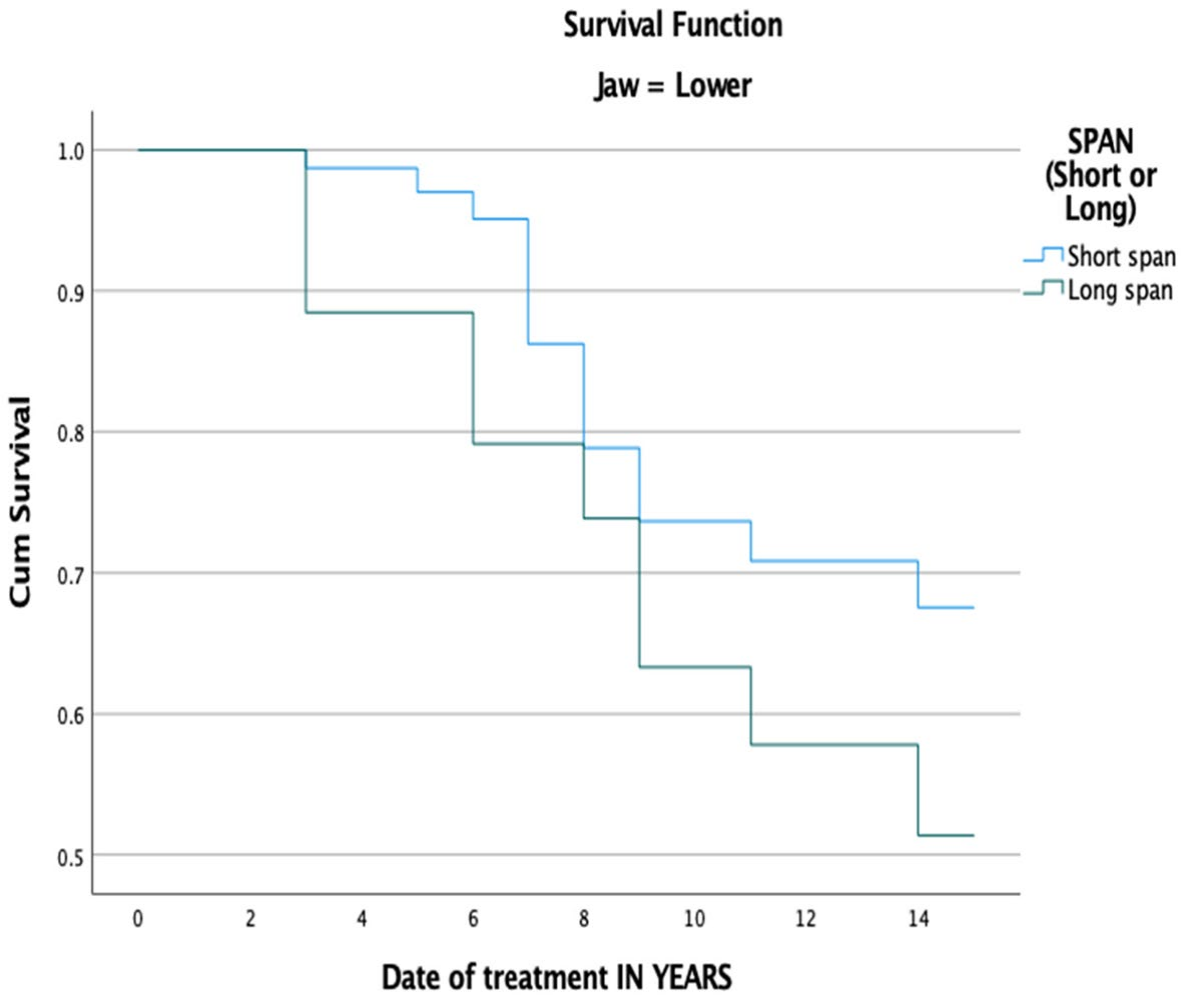
Kaplan–Meier survival function of FDPs in the lower jaw for primary outcome ‘technical complications’ based on span length, (*P*-value = .49)

Tables 4 and 5 show the life-table survival analysis of short- and long-span FDPs regarding the occurrence of technical complications. The cumulative survival rate for short-span FDPs was 91% in year 5, 68% in year 10, and 34% in year 15. For long-span FDPs, the cumulative survival rate was 85% in year 5, 50% in year 10, and 18% in year 15. The placement of short-span FDPs in the upper jaw revealed a significantly lower complication rate compared to long-span FDPs (*P* = 0.048) (Fig. 3). Meanwhile, there were no significant differences in complication rates between short- and long-span FDPs when placed in the lower jaw (*P* = 0.49) (Fig. 4).

**Table 4:** Life-table survival analysis showing the cumulative survival rate of FDPs in upper jaw when it comes to occurrence of technical complications

Interval Start Time (Years)	Number Entering Interval	Number Withdrawing during Interval	Number Exposed to Risk	**FDPs** Failure	Survival Rate within each Intrerval – ISR (%)	Cumulative Proportion Surviving at End of Interval	Std. Error (%)
1	141	13	134.500	4	0.97	0.97	0.01
2	124	7	120.500	5	0.96	0.93	0.02
3	112	15	104.500	0	1.00	0.93	0.02
4	97	9	92.500	2	0.98	0.91	0.03
5	86	15	78.500	2	0.97	0.89	0.03
6	69	3	67.500	3	0.96	0.85	0.04
7	63	3	61.500	5	0.92	0.78	0.04
8	55	4	53.000	4	0.92	0.72	0.05
9	47	2	46.000	1	0.98	0.70	0.05
10	44	9	39.500	7	0.82	0.58	0.06
11	28	2	27.000	0	1.00	0.58	0.06
12	26	1	25.500	0	1.00	0.58	0.06
13	25	1	24.500	1	0.96	0.56	0.06
14	23	0	23.000	0	1.00	0.56	0.06
15	23	15	15.500	8	0.48	0.27	0.08

**Table 5:** Life-table survival analysis showing the cumulative survival rate of FDPs in lower jaw when it comes to occurrence of technical complications

Interval Start Time (Years)	Number Entering Interval	Number Withdrawing during Interval	Number Exposed to Risk	**FDPs** Failure	Survival Rate within each Intrerval – ISR (%)	Cumulative Proportion Surviving at End of Interval	Std. Error (%)
1	117	8	113.000	0	1.00	1.00	0.00
2	109	10	104.000	4	0.96	0.96	0.02
3	95	13	88.500	0	1.00	0.96	0.02
4	82	7	78.500	1	0.99	0.95	0.02
5	74	9	69.500	3	0.96	0.91	0.03
6	62	7	58.500	4	0.93	0.85	0.04
7	51	2	50.000	4	0.92	0.78	0.05
8	45	1	44.500	4	0.91	0.71	0.06
9	40	0	40.000	0	1.00	0.71	0.06
10	40	5	37.500	2	0.95	0.67	0.06
11	33	1	32.500	0	1.00	0.67	0.06
12	32	1	31.500	0	1.00	0.67	0.06
13	31	1	30.500	2	0.93	0.63	0.06
14	28	1	27.500	0	1.00	0.63	0.06
15	27	18	18.000	9	0.50	0.31	0.08

## Discussion

The study evaluated the technical complications associated with FDPs over a long-term observation period. The examined patients were distributed somewhat equally between males and females. These may rule out the influence of patient gender on complications, particularly since no statistical differences were found (*P* = ,129).

The results showed that the majority of evaluated prostheses were short-span prostheses with 3 or 4 units (76.4%), which is understandable since long edentulous areas are usually treated with implant restorations or removable prostheses. The study showed that the estimated 5-year survival rate for short-span and long-span FDPs was 91%. This rate was close to what has been reported in a systematic review published by Pjetursson et al. [41] that showed a 93.8% survival rate for tooth-supported FDPs after 5 years. The same review reported a survival rate of 89.2% for tooth-supported FDPs after 10 years, which is better than the rates obtained in this study (the 10-year survival analysis was 68% for short span and 50% for long span) [41]. However, the survival rates presented in this study were based on the incidence of detecting technical complications and not necessarily losing the prosthesis. Furthermore, the findings from this study showed that the survival rate for both short- and long-span FDPs decreased more sharply after 10 years, which most likely due to long-term fatigue. Meanwhile, some researchers evaluated the longevity of short bridges and long bridges and reported that age of the patient did not influence the survival [42].

In the overall evaluation of short- and long-span FDPs, the findings from this study showed a statistically significant difference between the two groups (*P* = 0.003). In the literature, there has been no consistent view as to whether the length of the prosthesis (number of units) affects the long-term survival of FDPs. Näpänkangas et al. reported in an overall survival analysis after 10 years a significantly lower survival rate for long FDPs (5 or more units) compared to short bridges of 3 or 4 units [42]. The same finding was reported in another study by Reuter and Brose, who detected a higher tendency for failures in longer FDPs [22]. Some clinical investigations have suggested a clear relationship between the prosthesis’s number of units and its lifespan [43]. In a study published by Leempoel et al., 1,674 bridges were evaluated to analyze the influence of several factors on the survival rate of bridges [44]. They reported no significant difference in the survival rate among bridges of various lengths. Other studies have suggested no relationship between the prosthesis survival rate and the number of units [45].

With regard to the location of FDPs, most of the prostheses in this study were placed on posterior teeth. It is believed that there are more clinical indications to place FDP in the posterior region than in the anterior region. In their prospective cohort study, Schmitter et al., reported that long-span FDPs in the molar region can be associated with higher risks of failure than FDPs in the anterior region [42]. The anterior area is more esthetically demanding, which explains why bridge restoration may not be the first treatment option. Meanwhile, treatment with implant restoration may face limitations in the posterior area due to the presence of some vital structure or severe bone resorption following the loss of teeth. The survival rate of long-span FDPs was significantly lower for long-span bridges compared to short-span bridges when they were placed in the maxilla (*P* = ,048). In the maxilla, a lower survival rate for long-span FDPs compared to short-span FDPs was also reported in another retrospective study after 20 years of follow-up [34]. Meanwhile, no significant differences were found when the two groups were compared only in the mandible. However, other reports evaluating the failure of fixed prostheses in both arches found a higher incidence of failure in the maxilla compared to the mandible [46].

The majority of the evaluated FDPs were fabricated using PFM. This could be explained by the fact that most clinicians in previous decades preferred these materials during bridge design, since they combine strength and esthetics. Metal-ceramic FDPs have been used for many decades as the gold standard treatment option for replacing missing teeth. The zirconia-based FDPs have been investigated numerously in the last decade to improve their properties, especially from an esthetic perspective. Recently, zirconia has been used more frequently than metal-ceramics, as it offers good esthetics and lower prices [26]. However, some investigations have revealed more technical problems with zirconia-based FDPs than with metal-ceramic FDPs [47].

The large variation in numbers between PFM and all-ceramics seen in this study makes it very difficult to draw a conclusion with regard to the rate of complications between these materials. In their systemic review, Pjetursson et al. [28] investigated the failure rate of FDPs fabricated from different types of materials after 3 years of follow-up. The study revealed the highest failure rate for glass-infiltrated alumina FDPs (13.8%), followed by glass ceramic FDPs (10.9%) and zirconia ceramic FDPs (9.6%). Meanwhile, metal-ceramic FDPs had the lowest failure rate among all examined materials (5.6%) [28].

The findings from this study revealed a dramatic increase in the complication rate after 10 years of service, particularly with long-span FDPs. This could be a result of long-term fatigue and stress on dental materials. Stress fatigue in the oral environment, as well as function and parafunction, are all factors believed to affect the longevity of all-ceramic restorations. Stress fatigue is believed to have a great influence on the longevity of dental restorations [48]. Most of the technical complications detected in this study were ceramic fractures or chipping. In their clinical investigation, Pihlaja et al. [21] reported that the risks of ceramic chipping can increase with long-span FDPs of 5 units or more. A similar finding was reported by Sax et al. [49] in their clinical evaluation of zirconia-based FDPs after a 10-year observation period. Some long-term investigations have suggested that ceramic chippings on metal-ceramic FDPs happen more commonly during the first year in service but will slightly decrease after that [29]. In addition, ceramic chipping was seen as a minor complication that did not require replacement of the prosthesis and was usually detected by dentists during follow-up visits [35, 49]. Unfortunately, this is not the case with other complications, such as framework fractures, which mostly require prosthesis replacement. Some studies have reported that an increase in FDP length increases the risk of framework fractures [35, 50].

The results obtained from this retrospective study were based on the evaluation of previous treatments that were provided by several clinicians at different time periods. This method could be associated with sampling bias. Meanwhile, the long evaluation time (15 years) used during the survival analysis and the high number of patients (229 patients) that were examined may be seen as a strong aspect of this study. Some of the limitations of this study are that it did not investigate the history of the complications or the influence of other factors on the rate of complications, such as patients’ age and medical condition, or the existence of parafunctional habits, such as bruxism. Another limitation is that it only included conventional tooth-supported FDPs. Other types of FDPs, such as cantilever bridges or implant-supported FDPs, should be taken into consideration in future investigations. The vitality of the abutment teeth as a factor may also be investigated in the future.

Another limitation is that it did not contain similar numbers of FDPs between all the groups. Different groups should be evaluated based on ceramic type, such as zirconia. Including only the conventional type of FDP is another limitation. Other types of FDPs should be evaluated in future studies, such as cantilever bridges or bridges with many abutment teeth. More factors that should be evaluated in the future include the vitality of the abutment teeth and the amount of bone support.

## Conclusions

The long-span FDPs that consist of 5 units or more can be associated with higher risks of technical complications compared to short-span FDPs after long-term evaluation. The risks of technical complications with long-span FDPs were found to be greater in the upper arch than in the lower arch. Long-term controlled clinical studies are essential to confirm these findings.

## Supplementary Information

**Additional file 1.**

## Data Availability

The datasets used and/or analyzed during the current study are available from the corresponding author on reasonable request.
